# Self limited disorder in a young female with fever, abdominal pain and lymphadenopathy: a case report

**DOI:** 10.1186/1757-1626-2-9318

**Published:** 2009-12-14

**Authors:** Balaji Yegneswaran, Vishal Jain

**Affiliations:** 1 Department of Internal Medicine, Drexel University College of Medicine/St Peter's University Hospital Program, 254 Easton Ave, New Brunswick, NJ 08901, USA

## Abstract

Kikuchi-Fujimoto Disease is a rare, benign cause of lymphadenopathy that is often associated with fever, night sweats, and weight loss. The clinical and laboratory manifestation of Kikuchi-Fujimoto Disease are similar to those of lymphoma, tuberculosis, sarcoidosis, systemic lupus erythematosus, and it is often mistaken for these disorders. Definitive diagnosis is accomplished by lymph node biopsy. Awareness of Kikuchi-Fujimoto Disease among clinicians and pathologists is essential to avoid misdiagnosis and inappropriate treatment of patients with this self-limited disorder.

## Background

Kikuchi-Fujimoto disease is considered a benign, self-limited cause of cervical or generalized lymphadenopathy that usually remits spontaneously without recurring [1]. KFD was first described in young Japanese women, but has since been identified among patients of all ethnicities and ages [2]. As it is an uncommon and benign disorder, most clinicians do not consider KFD when evaluating patients with lymphadenopathy and fever. This can lead to unnecessary invasive testing and subsequent complications, as illustrated in our case. Through this case report, we aim to increase awareness of KFD as a benign entity that is often mistaken for other more serious illnesses.

## Case presentation

A 28-old Hispanic female presented to our hospital with abdominal pain and fever. She had been well until 3 weeks prior to admission, when she began experiencing night sweats and fevers. One day prior to admission she developed a constant, dull, non-radiating epigastric pain that was not alleviated or exacerbated by meals or medication. There was no recent history of nausea, vomiting, diarrhea, dysuria, rashes, arthralgias, or respiratory symptoms, but she did report a weight loss of approximately five pounds over the last 3 months.

Her past medical history revealed only an admission to the same hospital 4 months earlier for epigastric and right upper quadrant pain. At that time an ERCP was performed, and it showed no bile duct dilation or stones. However, she suffered severe pancreatitis following the procedure. She subsequently made a full recovery and was discharged home 22 days later.

Both of her parents are alive and healthy, with no history of malignancy in the family. According to the patient, she had a negative tuberculin skin test 5 years prior to admission and had no sick contacts. She had immigrated to the United State from Mexico nine years ago and had not traveled outside the country since then. She lives with her two healthy daughters in New Jersey and works as a homemaker. She was not taking any medications and had no known drug allergies.

At the time of admission, her temperature was 38.9°C, heart rate was 108, respiration rate was 16, and blood pressure was 102/52. She appeared thin but well nourished and was not in acute distress. The physical exam was remarkable for bilateral lymphadenopathy in the posterior cervical, supraclavicular, axillary, and inguinal regions. The nodes were firm, mildly tender, mobile, and up to 1.5 cm in diameter. The lungs were clear. Abdomen was soft, nondistended, and moderately tender to palpation in the right upper quadrant and epigastric region. There was no rebound or guarding, and Murphy's sign was negative. Stool was negative for occult blood. Skin examination revealed no rashes.

The patient's initial laboratory values are presented in Table 1. CT of the abdomen showed no evidence of acute pancreatitis, but revealed para-aortic, aortocaval, and right lower quadrant lymphadenopathy characterized as "stable" compared to a CT during the previous admission. CT of the thorax revealed bilateral pleural effusions, scattered peripheral ground glass parenchymal densities, several sub-centimeter nodular densities in the right lung base, and prominent lymph nodes in the perivascular space and in the precarinal region. No hilar adenopathy was noted. MRCP of the abdomen showed no evidence of biliary ductal dilation.

**Table 1:** Initial laboratory values

	Patient value	Reference range
WBC	**3.6**	4 - 11 per mm^3^
Hemoglobin	**9.5**	12 - 16 per dL
Hematocrit	**28.1**	35 - 47%
Platelets	**163**	150 - 400 per mm^3^
Neutrophils	85	37 - 75%
Lymphocytes	**7**	12 - 50%
Sodium, serum	**131**	136 - 145 mmoL per L
Potassium, serum	**3.3**	3.5 - 5.1 mmoL per L
Chloride, serum	97	99 - 112 mmoL per L
Bicarbonate, serum	25	21 - 33 mmoL per L
Creatinine, serum	0.4	0.6 - 1.2 mg per dL
Blood urea nitrogen, serum	6	9 - 28 mg per dL
Glucose, serum	86	82- 115 mg per dL
Calcium, serum	8	8.4 - 10 mg per dL
ALT	**142**	9 - 52 units per L
AST	**352**	14 - 36 units per L
Amylase, serum	148	30 - 151 units per L
Lipase, serum	51	23 - 300 units per L
Alkaline Phosphatase, serum	**266**	56 - 119 units per L

The patient was treated initially with imipenem/cilastatin. However, she continued to spike low-grade temperatures during her stay in the hospital. She never appeared toxic or acutely ill, and she tolerated a regular diet. Her WBC count fell to 2.2 and then rose to 4.6 by the fifth hospital day. Her hemoglobin during the same time fell to 8.5, and then peaked at 10.2 without transfusion. Folate and vitamin B12 levels were normal, as was the MCV. The Ferritin level was elevated to 17,636, the haptoglobin was high at 344, and the ESR was 97. Serum lactate dehydrogenase concentration was elevated above 5000 for the duration of her hospitalization. The ANA, anti-smooth muscle antibody, anti-mitochondrial antibody, HIV screening panel, two sets of sputum acid-fast stains and cultures, two sets of blood cultures, and hepatitis A, B and C viral antibody tests were all negative.

**Figure 1:**
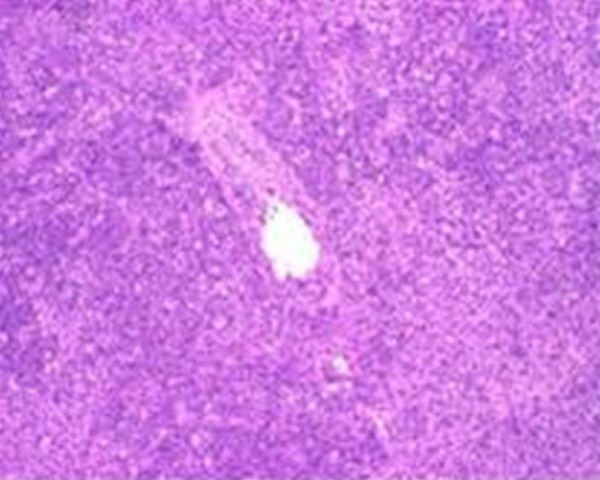
**Supraclavicular lymph node: low power magnification shows numerous lympocytes and irregular eosinophilic area of necrosis**.

**Figure 2:**
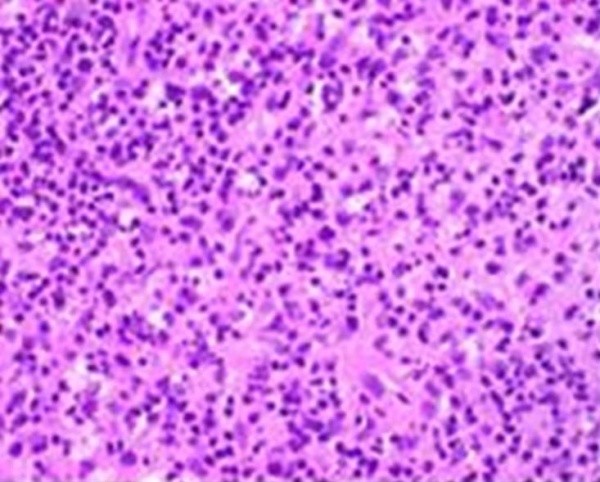
**Supraclavicular lymph node: high power magnification reveals histiocytes and karyorrhectic debris**.

Biopsy of a supraclavicular lymph node revealed variable size eosinophilic areas containing histiocytes, lymphocytes, immunoblasts, karyorrhectic and eosinophilic granular debris consistent with Kikuchi-Fujimoto disease (Figure 1 and Figure 2). Flow cytometry and immunostaining were negative for markers of lymphoma, and special stains for mycobacteria and fungi were negative. Antibiotics were discontinued, and the patient was discharged home with instructions to return to clinic for follow-up. She was counseled regarding the benign nature of her disease. At follow-up one month later, the patient was doing well, yet she continued to experience sporadic low-grade fevers.

## Discussion

Kikuchi-Fujimoto Disease (KFD) is a rare, benign, self-limiting cause of lymphadenopathy first described in 1972 [1,3]. It typically presents with non-tender or mildly tender enlargement of the posterior cervical or supraclavicular lymph nodes. Involvement of parotid, axillary, inguinal, mediastinal, and mesenteric nodes has also been reported. Generalized lymphadenopathy is an uncommon manifestation of KFD, but it can occur [4].

Historically, it has been reported that KFD affects women more than men in a ratio of approximately four to one [5-7]. Recent reports seem to indicate that the female preponderance was overemphasized in the past, and that the actual ratio is closer to 1:1 [4,7]. Though it was originally described in East Asian patients, it has since been recognized virtually worldwide. The mean age at presentation is 30 years. Signs and symptoms commonly associated with KFD include fever, night sweats, weight loss, and less commonly, rash, arthralgias, sore throat, nausea, vomiting, and abdominal pain. The cause of KFD is unknown. Various theories have been proposed. One theory is that KFD represents the late sequelae of infection by an associated pathogen. Pathogens that have been implicated include Human Herpes Viruses 6 and 8, Herpes Simplex Virus, Adenovirus, Parvovirus B19, Epstein Barr Virus Cytomegalovirus, Varicella Zoster Virus, Dengue Virus, M. Azulgai, Yersinia sp., and protozoa [8,9]. In addition, familial occurrences have been reported, and thus a genetic predisposition has been suggested. However, none of these etiologies has been confirmed, and the basic pathophysiology of KFD remains unclear.

Definitive diagnosis of KFD is made by lymph node biopsy. In the past, FNA has been utilized for diagnosis, but the results are often equivocal because the nodal architecture cannot be appreciated adequately. Pathological evaluation of the excised node characteristically reveals patchy or confluent areas of paracortical necrosis with karyorrhectic areas formed by histiocytes, plasmacytoid monocytes, and immunoblasts. There is a notable absence of neutrophils and eosinophils [10]. Laboratory abnormalities that can occur in KFD include anemia, leukopenia or even pancytopenia, elevated erythrocyte sedimentation rate (ESR), and elevated lactate dehydrogenase (LDH). Currently there is no laboratory test specific for KFD.

KFD is a benign, self-limited disorder, and symptoms usually resolve spontaneously over several weeks to months. Several authors have reported favorable responses to corticosteroid treatment for severe cases, but therapeutic measures are generally limited to patient education and symptomatic treatment with NSAIDs. Close follow up of KFD patients is indicated in order to confirm the resolution of their symptoms within the usual time course, and also to assess for signs suggesting the development of systemic lupus erythematosus (SLE). The two diseases share similar hosts and similar manifestations, and there have been reports of KFD patients going on to develop SLE [5]. Therefore patients need to be followed closely even after all symptoms of KFD have resolved.

Since the clinical presentation and laboratory findings seen in KFD overlap notably with those of lymphoma, tuberculosis, sarcoidosis, and systemic lupus erythematosus, yet its treatment differs greatly, differentiating KFD from these conditions is essential. Lack of awareness of KFD among clinicians and pathologists has resulted in unnecessary treatments with chemotherapeutics and high dose steroids. Therefore, KFD should always be considered in the differential diagnosis of lymphadenopathy and fever.

## Abbreviations

ESR: erythrocyte sedimentation rate; KFD: Kikuchi-Fujimoto Disease; LDH: lactate dehydrogenase; SLE: systemic lupus erythematosus.

## Consent

We are unable to gain consent for publication, all reasonable attempts to gain consent have been made the patient is anonymous there is no reason to think that the patient or their family would object to publication.

## Competing interests

The authors declare that they have no competing interests.

## Authors' contributions

BY primary resident in the management of the case, manuscript preparation. VJ was primary attending on the management of the case, manuscript reviewing and editing. Mentorship in writing the case report.

All authors read and approved the final manuscript.

## References

[B1] Kikuchi M Lymphadenitis showing focal reticulum cell hyperplasia with nuclear debris and phagocytes: a clinicopathological study Acta Hematol Jpn 1972 35 379 380

[B2] Ramanan AV Wynn RF Kelsey A Baildam EM Systemic juvenile idiopathic arthritis, Kikuchi's disease and haemophagocytic lymphohistiocytosis--is there a link? Case report and literature review Rheumatology 2003 42 4 596 598 10.1093/rheumatology/keg167 12649409

[B3] Fujimoto Y Kozima Y Yamaguchi K Cervical subacute necrotizing lymphadenitis: a new clinicopathologic entity Naika 1972 20 920 927

[B4] Bosch X Guilabert A Miquel R Campo E Enigmatic Kikuchi-Fujimoto disease: a comprehensive review Am J Clin Pathol 2004 122 1 141 152 10.1309/YF081L4TKYWVYVPQ 15272543

[B5] Dorfman RF Berry GJ Kikuchi's histiocytic necrotizing lymphadenitis: an analysis of 108 cases with emphasis on differential diagnosis Semin Diagn Pathol 1988 5 4 329 345 3217625

[B6] Asano S Akaike Y Jinnouchi H Muramatsu T Wakasa H Necrotizing lymphadenitis: a review of clinicopathological, immunohistochemical and ultrastructural studies Hematol Oncol 1990 8 5 251 260 10.1002/hon.2900080503 2249796

[B7] Lin HC Su CY Huang CC Hwang CF Chien CY Kikuchi's disease: a review and analysis of 61 cases Otolaryngol Head Neck Surg 2003 128 5 650 653 10.1016/S0194-5998(02)23291-X 12748557

[B8] Huh J Kang GH Gong G Kim SS Ro JY Kim CW Kaposi's sarcoma-associated herpesvirus in Kikuchi's disease Hum Pathol 1998 29 10 1091 1096 10.1016/S0046-8177(98)90419-1 9781647

[B9] Yen A Fearneyhough P Raimer SS Hudnall SD EBV-associated Kikuchi's histiocytic necrotizing lymphadenitis with cutaneous manifestations J Am Acad Dermatol 1997 36 342 346 10.1016/S0190-9622(97)80413-6 9039215

[B10] Kung IT Ng WF Yuen RW Chan JK Diagnosis by fine needle aspiration Acts Cytol 1990 34 323 2343686

